# RandAgiamo™, a Pilot Project Increasing Adoptability of Shelter Dogs in the Umbria Region (Italy)

**DOI:** 10.3390/ani5030383

**Published:** 2015-08-14

**Authors:** Laura Menchetti, Stefania Mancini, Maria Chiara Catalani, Beatrice Boccini, Silvana Diverio

**Affiliations:** 1 Department of Veterinary Medicine, Perugia University, Via San Costanzo 4, 06126 Perugia, Italy; E-Mail: lallymen@libero.it; 2 Public Veterinary Services for Urban Hygiene and Prevention of Stray Dogs, USL Umbria 1, Municipal Rescue Dog Shelter, Strada per Brufa snc, 06148 Collestrada (Perugia), Italy; E-Mail: stefania.mancini@uslumbria1.it; 3 Veterinary Consultant, 06100 Perugia, Italy; E-Mails: mchiaracatalani@gmail.com (M.C.C.); beabocc@yahoo.it (B.B.); 4 Laboratory of Ethology and Animal Welfare (LEBA), Department of Veterinary Medicine, Perugia University, Via San Costanzo 4, 06126 Perugia, Italy

**Keywords:** shelter dogs, adoption rate, human-dog interaction, training, RandAgiamo project, no-kill policy

## Abstract

**Simple Summary:**

In Italy, dog shelters are overcrowded because the rate of dog adoption is lower than that of abandonment. A project called “*RandAgiamo*” was implemented in a rescue shelter in central Italy. RandAgiamo provides training, socialization and advertising of adult shelter dogs. Official data of the Umbria regional health authorities from the year 2014 showed a higher rate of adoption in shelters involved in the project. RandAgiamo dogs had triple odds of being adopted compared to others housed in shelters of the same province. The increase in adoption rate can be beneficial for both dog welfare and shelter management.

**Abstract:**

Current Italian legislation does not permit euthanasia of dogs, unless they are ill or dangerous. Despite good intentions and ethical benefits, this “no-kill policy” has caused a progressive overpopulation of dogs in shelters, due to abandonment rates being higher than adoption rates. Shelter overcrowding has negative implications for dog welfare and increases public costs. The aim of this paper is to describe the pilot project “*RandAgiamo*” implemented in a rescue shelter in the Umbria Region and to evaluate its effectiveness on the rate of dog adoption using official data. RandAgiamo aimed to increase adult shelter dogs’ adoptability by a standard training and socialization programme. It also promoted dogs’ visibility by publicizing them through social media and participation in events. We analysed the official data of the Umbria regional health authorities regarding dog shelters of the Perugia province of the year 2014. In the RandAgiamo shelter, the dog adoption rate was 27.5% higher than that of dogs housed in other shelters located in the same geographical area (*p* < 0.001). The RandAgiamo project could be beneficial for the dogs’ welfare, owner satisfaction, shelter management, and public perception of shelter dogs. However, staff were required to provide dog training and related activities.

## 1. Introduction

It is recognised that dogs living in shelters are mostly the outcome of a break of the human–animal bond [1]. In Italy, Law No. 281 “Framework law on animal disease and prevention of stray dogs”, approved in 1991, introduced a national “no-kill policy” for dogs, unless they are suffering from incurable diseases or are proven to be dangerous to human society [2]. As a result, the fate of abandoned dogs completely changed: if not claimed by their owners within three days of being captured by authorities, they could no longer be euthanized, instead dogs have to be housed in adequate shelters until adopted. Unfortunately, for some dogs this means living in a confined space for the duration of their lives. Countries such as Austria have similar provisions, while in many others, such as USA and Australia, euthanasia is still a routine practice to control overpopulation. Although there are no official statistics, estimates reported in these countries suggested a euthanasia rate of 30%–60% [3,4,5,6]. The Italian law No. 281/1991 made sterilization of shelter dogs compulsory, as well as the registration and identification of all owned dogs. The ultimate aim of this legislation was the transformation of dog shelters into a “temporary place of transition”, from straying to returning to the owner or to being adopted.

Notwithstanding the ethical intent of the “no-kill policy” in Italy, after 25 years this law has had negative implications. As a result, there has been (i) an increase in the number of dogs housed in shelters, due to rate of adoption being lower than that of abandonment; (ii) an increase in public costs to fund the shelters; and (iii) a reduction in dog welfare, when poor shelter management and/or overcrowding is not conducive to an acceptable standard of care [7,8].

Recent data suggests there are currently 6 million owned dogs and 590 thousand stray dogs in Italy, of which only one third are housed in shelters [9]. It has been estimated that the number of dogs that entered Italian shelters amounted to 104,000 in 2011 [10], while pets in Italian homes decreased from 42% in 2012 to 33% in 2015 [11]. A recent survey reported an increase in the number of people trying to foster their pets in other families while the propensity to adopt was decreasing [12]. Moreover, a study in a public shelter located in Northern Italy showed that about 15% of the adopted dogs were re-relinquished to the shelter [1]. Behavioural problems were the most frequent causes of return [1].

In Italy, there is currently a great debate among legislators, both at national and regional levels, to find solutions to reduce the number of dogs in shelters. One of the most important unsolved problems is the reticence of Italian owners to microchip their dogs and enrol them in the National Dog Register Service. This lack of compliance by a large number of dog owners has dramatic consequences not only on their dogs, but also on society as a whole. Abandoned dogs become free roaming, wandering around human living areas looking for food and protection. Stray dogs may become a vehicle of infectious disease, cause road accidents, cause damage to livestock and wild animals, and represent a potential risk of aggression to people [9]. Healthy captured dogs, which are not registered, cannot be returned to their owners or euthanized and subsequently the number of dogs housed in shelters continues to increase.

Capture operations and confinement to a shelter has been shown to be one of the most significant stressors a companion dog can experience, potentially decreasing welfare status [13,14,15,16,17,18]. It has been shown that social and spatial restriction, the main stressors in a shelter, result in increased excitement, aggression, and uncertainty [15]. Behavioural indicators of stress are accompanied by increased cortisol secretion and altered responsiveness of the pituitary–adrenal axis [16]. Many dogs show signs of depression in the first weeks after entering a shelter [17]. The abandonment and separation distress of the dog is related to the strength of the attachment bond with the lost owner [18]. Living in a shelter may promote stress related behaviours in dogs that could be undesirable by people, making dogs less adoptable [14].

In Italy, there is a widespread public perception that dog shelters are merely dumping grounds for the most ugly and badly behaved dogs. This undesirable image acts to perpetuate the low adoption rate of dogs in shelters. Many strategies have been implemented to promote dog adoptability. Among these, dog training and socialization programs have proven to be very successful, greatly improving the quality of life of sheltered dogs [18,19,20]. However, further studies are needed because although training and human interaction may function as enrichment interventions for shelter dogs, in another study these interventions have proved ineffective in producing cost-efficient increases in adoption rates [21].

In a rescue shelter in central Italy (Collestrada, Perugia), a pilot project called “*RandAgiamo*” has been implemented. RandAgiamo aimed to increase the adoptability of adult shelter dogs by a standardized socialization and training protocol. The project also organised a campaign to raise public awareness and promote dogs’ adoption through social media and participation in public events.

The aim of this paper is to evaluate the effects of the RandAgiamo project on shelter dogs’ adoption rates by using official data of the Umbria regional health authorities collected in 2014.

## 2. Materials and Methods

### 2.1. The “RandAgiamo” Project: Philosophy and Activities

The RandAgiamo Project was implemented in 2010 as a pilot study at the Municipal Rescue Dog Shelter of Collestrada (Perugia) to explore new solutions to increase adoption rates of shelter dogs in the Umbria Region. It was a collaborative project between the Laboratory of Animal Ethology and Welfare (LEBA) of the Department of Veterinary Medicine of Perugia University, and the Public Veterinary Services for Urban Hygiene and Prevention of stray dogs of USL Umbria 1 (PVS-USL Umbria1).

In 2013 the Umbria Region, within the framework of the 2011–2012 Regional Prevention Plan (DGR n. 1253/2011), promoted the implementation of the RandAgiamo Project as an operational model to be extended to other public dog shelters in the Umbria Region. During the first three years of the project (2010–2012), the efficacy of the protocol was tested by assessing its effectiveness on dog behaviour, and the benefits of post-adoption assistance to new dog owners [22,23,24,25]. At the end of the three-year period, we carried out a survey, by telephone interview of the adopters to verify the degree of their satisfaction and if the RandAgiamo dogs transferred the learned competences to daily family life [23]. The year 2013 was a transition period for the implementation of the RandAgiamo project, which aimed to integrate all the procedures and protocols into shelter management operations.

The word RandAgiamo (a registered trade mark, protocols and procedures are under registration) underlines the willingness to do something actively for the love of lost dogs (Randagi + Amo = *free roaming dogs + love*; Agiamo = *Let’s do something!*). RandAgiamo aimed to improve shelter management of dogs and facilitate adult shelter dogs’ relationships with new adoptive families. To increase adoptability, a standardized training protocol was implemented to encourage social activities of the dogs and to promote dog welfare through environmental enrichment. Through this socialization program and by certifying dogs as well trained and safe companions, it was hoped that the quality of life of shelter dogs would improve and there would be a change to the public image of shelter dogs.

RandAgiamo staff included LEBA personnel, in close collaboration with PVS-USL Umbria1 personnel (shelter veterinarians and technicians). The RandAgiamo staff included two veterinary behaviourist and a clinician veterinarian, eight RandAgenti (dog training operators), and about 50 volunteers for dog walking. All the RandAgiamo staff were trained by specific courses before commencing the project activities. The basic training course consisted of three days of theory concerning dog behaviour, learning and training techniques, dog communication and welfare, stray dog problems and shelter management. Most of the RandAgenti (*n* = 6) were already qualified as dog trainers; the others (*n* = 2) were students in veterinary medicine, with an internship at the LEBA. In addition to the three-day basic training course, the RandAgenti attended an additional practical phase according to their personal predisposition and working ability with dogs (only positive reinforcement (food and praise) was used for dog training). This stage varied from one to three months. During the practical stage, each new RandAgente (a single dog training operator) was specifically trained by one behaviourist veterinarians of the RandAgiamo staff or was put side-by-side and supervised by an already well-trained RandAgente. Once the supervisor and the RandAgiamo personal training facilitator agreed the “in training RandAgenti” could train a RandAgiamo dog on their own, these could be fully involved with the dog training activities. Similarly, the volunteers attended the basic training course and were practically trained by a RandAgente. Only once the RandAgente considered the “in-training volunteers” able to walk the dogs safely outside the shelter on their own could the volunteers be independent.

A minimum participation rate was required by volunteers—at least twice a month for a period of six months. However, volunteers freely participated much more frequently, so that most of the year dogs were walked outside up to three times a week. To avoid attachment problems between dogs and the RandAgenti and volunteers, each dog was always trained and walked by different staff. Overall, during 2014, the RandAgenti behaviourally assessed a total of 70 dogs, fully training 58 of them. In the RandAgiamo shelter, RandAgenti and volunteers also included some untrained dogs in the daily dog walk. Most of the RandAgiamo staff worked on a voluntary basis, which made this program economically affordable.

**Figure 1:**
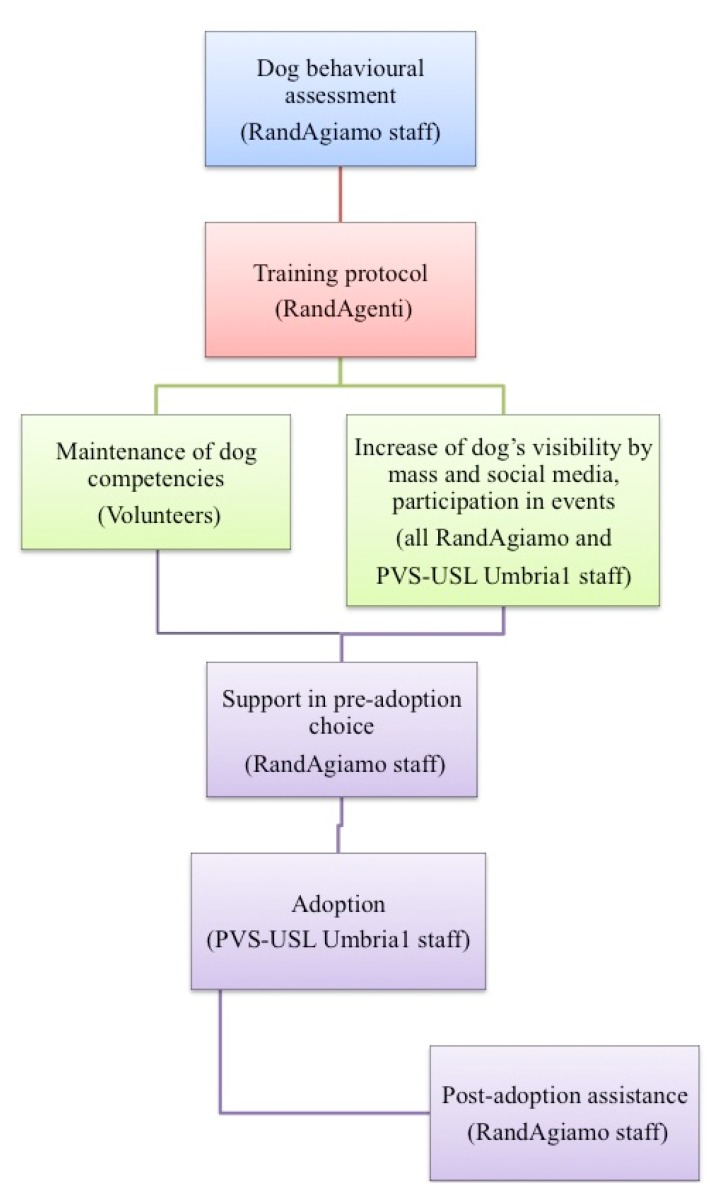
RandAgiamo Project flow chart.

The standardized training protocol of the RandAgiamo project included different elements and procedures (Figure 1).

Only dogs older than an estimated age of eight months were included in the project. However, adult and elderly dogs were preferentially chosen, due to their low adoptability [1,3,14]. Before entering into the RandAgiamo training protocol, every dog was behaviourally assessed according to a predefined behaviour check point system [22,25]. This behavioural assessment method was based on a 5 Likert score system measuring some behavioural characteristics such as sociability, fear, excitability, *etc*. This procedure is being validated [25]. If a dog was judged suitable it was started on the socialization and training programme.

**Figure 2:**
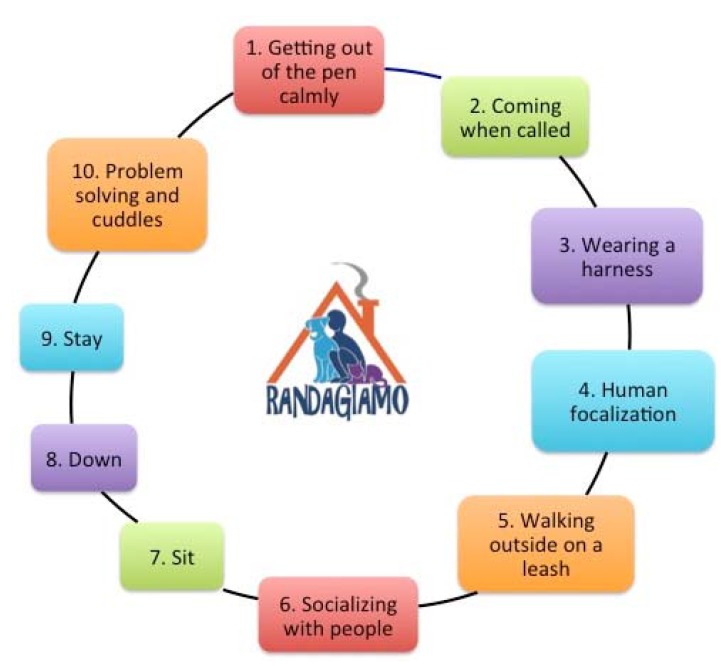
RandAgiamo training protocol: the 10 steps of each session.

The complete training protocol consisted of six sessions carried out over a period of two weeks (three sessions/week). Each session included a predefined series of 10 steps (training exercises): getting out of the pen when calm; coming when called; wearing a harness; human focus; walking outside on a leash; socializing with people; responding to commands “sit, down, stay”; problem solving and relaxing with cuddles (Figure 2). For each step, a maximum time of 5 min was allowed, with the exception of walking outside on a leash, which was up to 15 min. All the steps of the RandAgiamo operational training were planned and performed according to dog ethology and welfare principles. All dogs that completed all the sessions of the standardized training protocol were named “*RandAgiamo dogs*”.

In the RandAgiamo shelter, potential adopters were supported to choose a dog by shelter and RandAgiamo staff. Adopters’ family and personal needs and situation were always considered to help match the right dog with the right home. At times, adopters were attracted to the shelter through the publicity of RandAgiamo dogs but in the end opted for an untrained dog. For ethical reasons, and in accordance with the RandAgiamo philosophy that all shelter dogs needed to be adopted and find a family, there was no discouragement of adopting any dogs, either trained (RandAgiamo dogs) or untrained.

RandAgiamo also provided post-adoption assistance to new adopters. A free consultancy with an expert behaviourist veterinarian was offered if they had any problems during the transition from shelter to inclusion in the family (Figure 1). The RandAgiamo project guaranteed the education of the dog and the assistance to the adopters so as to prevent the return of adopted dogs to the shelter or their abandonment.

The project implemented a campaign to raise public awareness and promote shelter dogs’ visibility through mass media: local newspapers, television and online social networks, and through participation in events (Figure 1). For example, a local newspaper regularly published a whole page dedicated to the human–animal relationship with articles written by RandAgiamo staff. Each time, the project was promoted and two different RandAgiamo dogs were advertised for adoption. This page was published biweekly throughout 2014. The page called “La casa degli animali” (The animal home) dealt with different topics regarding pet behaviour and welfare, curiosities and things worth knowing about animal life. This was a wonderful opportunity to educate the public about the ethological and physiological needs of companion animals and raise awareness of the problem of stray dogs. In addition, an advertising newspaper published photographs and individual information about up to two RandAgiamo dogs every week. The same ad was repeated a few times. “Progetto RandAgiamo” and “Sportello a 4 Zampe—Provincia di Perugia” Facebook pages promoted the project activities and appealed to the public to adopt shelter dogs. A local television appearance was made four to five times a year to promote the RandAgiamo project. A number of events were organized at the shopping centre nearby the RandAgiamo dog shelter. Here, the RandAgiamo dogs, always under the protection and guide of the RandAgiamo staff, interacted with people outside of the shelter, entering into the daily life of the general public.

### 2.2. Animals and Housing

Data was obtained from two Municipal Dog Shelters located at Collestrada (Perugia, Italy): “RandAgiamo”, the rescue shelter [26] involved in the project and “Control”, the corresponding rehoming shelter [27] located nearby (less than 1 km away). In both structures, the same Animal Protection Organization provided dog husbandry (daily diet and cleaning operations). We selected the latter shelter to act as the “control” because husbandry was managed by the same organization, and it was located in the same geographic area.

The RandAgiamo and Control shelter comprised different sized pens, and in each one of them a variable number of dogs could be housed. Variability in pen size and dog number is a management tool, which allows the grouping of dogs according to their temperament and socialization. Only shelter personnel responsible for the daily care of the animals were able to make the choice of how to house an individual dog. Dogs were housed in a pair or in small groups (from three to up 12 dogs) within fenced areas (pens) (size ranged between 15 and 40 m^2^) or dog cages (average size of 3 m × 1.5 m). Each pen was provided with a covered area, where dogs could shelter from the sun or the rain. Aluminium or plastic bowls for water and food were provided for each animal. Dogs were fed once a day (between 8.00 am and 10.00 am) with commercial dry food, occasionally supplemented with wet food (bread, pasta and dog meat). Cleaning and cage management were carried out daily, between 6.00 am and 1.00 pm, even on weekends. Each dog had a kennel inside the pen, but often they preferred to sleep next to each other. In the RandAgiamo shelter, no other inanimate enrichments were present.

Some aspects varied between the RandAgiamo and Control shelters, such as animal health and daily management. In the RandAgiamo shelter, health management was provided by the Public Veterinary Services of the USL Umbria 1 that implemented the project. Conversely, in the Control shelter, the Animal Protection Organization managed the shelter health. In the RandAgiamo shelter, the dogs were managed according to the project philosophy. Here, each shelter dog was guaranteed 1–6 h per day to roam free in an outdoor common space within the shelter, together with dogs living in different groups. Dogs were also moved into this area during their pen cleaning. Dogs selected for the RandAgiamo training protocol were also walked outside the shelter on a countryside path (during the training or the walks with the RandAgenti and volunteers). In the Control shelter, the dogs were never allowed to go outside the shelter to be walked, but they could in turn walk in adjacent fenced areas just outside their pens.

Rescue and rehoming data from other shelters of the Perugia province were also evaluated: Città di Castello, Gubbio, Assisi and Todi. These shelters had great variation in husbandry practices, housing conditions, grouping and daily management. Animal health was always managed by the USL Umbria Public Veterinary Services in the RandAgiamo and Control shelters, whilst animal husbandry was managed by private animal protection organizations in both rescue and rehoming structures.

### 2.3. Data Collection and Processing

The Sanitary Agency of the Umbria Region, USL Umbria1, provided data for the year 2014 for the RandAgiamo and Control shelter, as well as for overall provincial data. This latter data refers to the 10 dog shelters present in the province: five rescue shelters (including the RandAgiamo one) and the five corresponding rehoming shelters (including the Control shelter). Official data were obtained by consulting the National Dog Register Service (Table 1). This source might pose a limit to the scientific insight of the paper due to the lack of specific and detailed data (*i.e.*, dogs’ sex, age, breed, length of stay, post-adoption returns, *etc.*) as well as of the adoption rate of years prior to implementation of the programme (*i.e.*, prior to 2010). Nonetheless it enabled an objective discussion of project outcomes. Furthermore, the official data of the Umbria regional health authorities are reported annually to the Italian Ministry of Health and used to assess action areas and management of the funds for stray dogs of the Italian Regions. The data selected for analysis in this paper are the same that inform the political decisions of the Italian Ministry of Health for each Region.

On 1 January 2014, a total number of 140 dogs were housed in the RandAgiamo shelter, 467 dogs in the Control shelter (Table 1). Overall, 187 and 1172 were housed in the rescue and rehoming shelters of the Perugia province, respectively (Table 1).

**Table 1:** Data for the year 2014 for the RandAgiamo and Control shelters, and Perugia province overall, provided by the Sanitary Agency of the Umbria Region, USL Umbria1.

		Dogs				
		Present at 1 Jan 2014	Entered during year 2014	Born in Shelter	Returned to Owner	Dead in Shelter
**Shelter**						
	**RandAgiamo**	140	546	0	294	10
	**Control**	467	198	N/A ^1^	N/A ^2^	59
**Perugia province**						
	**Rescue shelters ^3^** (*n* = 5)	187	1199	4	450	26
	**Rehoming shelters ^4^** (*n* = 5)	1172	474	N/A ^1^	N/A ^2^	110
	**Total ^3,4^** (*n* = 10)	1359	1673	4	450	136

^1^ Not applicable because all dogs are sterilised; ^2^ Not applicable because dogs are usually returned to their owner within 60 days after capture, and within this period they are compulsorily kept in a rescue shelters (see [26,27], and Supplementary Material); ^3^ Including the RandAgiamo shelter; ^4^ Including the Control shelter.

The *total numbers of dogs* that were housed at least one day in each shelter in the year 2014 were calculated as the sum of dogs present in the structure as at 1 January 2014, those who entered and those who were born during that year.

*Dogs available for adoption* in the year 2014 were calculated by subtracting from the total number of dogs in each shelter, the number of dogs identified by the Registration Dog Service and returned to their owner, and the number of dead dogs in that year.

*The adoption rate* was calculated as a ratio between the adopted dogs and those available for adoption dogs.

Adoption rate = Dogs adopted / Dogs available for adoption

*The restitution rate* was calculated as the ratio between the number of dogs returned to their owners and those entering the rescue shelter.

Restitution rate = Dogs returned to their owners / Dogs entered shelter

### 2.4. Statistical Analysis

Data were analysed by using Chi Square (χ^2^). Cramer’s V (V) was also reported as a measure of the strength of association not sensitive to sample size. Binary logistic regression procedure was used to calculate the odds ratio, 95% confidence interval (95% CI), and P value of Wald statistic (SPSS statistical version 20.0 software package, USA).

## 3. Results

The adoption rate of RandAgiamo was 27.5% higher than Control shelter (χ^2^ = 76.11, *p* < 0.001; V = 0.278; *p* < 0.001): 53.9% of dogs within the RandAgiamo shelter were adopted, while the adoption rate of the Control shelter was 26.4% (Table 2).

**Table 2:** Cross-tabulation of the number of no-adopted *versus* number of adopted dogs in the RandAgiamo and the Control shelter.

	Shelter		Total
	RandAgiamo	Control	
**No adopted**	176_a_	446_b_	622
**Adopted**	206_a_	160_b_	366
**Total**	382	606	988

Each subscript letter denotes a subset of shelter categories whose column proportions do not differ significantly from each other at the 0.05 level.

The adoption rate of all rescue and rehoming shelters of the Perugia province, but not including the RandAgiamo shelter, was 28.9%. The adoption rate of the RandAgiamo shelter was significantly higher than all other shelters (53.9% *vs.* 28.9%; χ^2^ = 91.48; *p* < 0.001; V = 0.193; *p* < 0.001).

In the RandAgiamo shelter, the odds that a dog was adopted was 3.3 times the odds in the Control shelter (95% CI = 2.5–4.3; *p* < 0.001) and 2.9 times the odds of others shelters in the same province (95% CI = 2.3–3.5; *p* < 0.001).

Considering the overall Perugia province data, the adoption rate was higher in rescue shelters (45.8%, including the RandAgiamo one) compared with rehoming ones (25.1%; χ^2^ = 112.20, *p* < 0.001; V = 0.214; *p* < 0.001). However, the RandAgiamo adoption rate was higher also compared to that of other rescue shelters (53.9% *vs.* 40.0%; χ^2^ = 131.66, *p* < 0.001; V = 0.193; *p* < 0.001; Table 3). Overall, the number of dogs available for adoption was 2450 and the adoption rate of the Perugia province in 2014 was 32.8% (Table 3).

The restitution rate was significantly greater in the RandAgiamo shelter (294 dogs were returned to their owners out of 546 captured dogs, 53.8%) compared with other rescue shelters of the Perugia province (156/653; 23.9%; χ^2^ = 113.82, *p* < 0.001; V = 0.232; *p* < 0.001).

**Table 3:** Cross-tabulation of the number of non-adopted *versus* the number of adopted dogs in the RandAgiamo shelter, all other rescue shelters and rehoming shelters (including the Control) of the Perugia province. Numbers inside the brackets show the percentage within each shelter category.

	Shelters			Total
	RandAgiamo	Other Rescue	Rehoming	
**Non adopted**	176 (46.1%)_a_	319 (60.0%)_b_	1151 (74.9%)_c_	1646 (67.2%)
**Adopted**	206 (53.9%)_a_	213 (40.0%)_b_	385 (25.1%)_c_	804 (32.8%)
Total	382 (100.0%)	532 (100.0%)	1536 (100.0%)	2450 (100.0%)

Each subscript letter denotes a subset of shelter categories whose column proportions do not differ significantly from each other at the 0.05 level.

## 4. Discussion

This study examined the effects of the RandAgiamo protocol on the adoption rate of adult shelter dogs. Through comparison with dog shelters managed by the same Animal Protection Organization and others located in the same geographic area, it was found that in the RandAgiamo shelter the adoption rate was increased and the dogs had triple odds of being adopted. This can bring tangible benefits:

(1) for the shelter, because an increase in the adoption rate reduces the number of housed dogs, improving management and reducing public costs;
(2) for the dogs, because a higher probability of being adopted increases the possibility of improved welfare, since living in a family, rather than in a pen, is more respectful of the social nature of the dog.

Official data provided by the Umbria regional health authorities posed some limits to this study due to the lack of specific data and adoption rate of the years prior to implementation of the programme. However, they enabled an objective discussion of project outcomes. The RandAgiamo philosophy assumed that interaction with humans and training could improve dog behaviour and make shelter dogs more attractive to the general public for adoption. The success of the adoption rate recorded seems to support this hypothesis, despite that other project elements could have contributed to it, such as publicity and pre-adoption assistance. In previous research, it was found that with time the RandAgiamo training dogs acquired more confidence and familiarity with all the procedures and social and environmental stimuli they were faced with [22,23,24]. They showed a calmer demeanour, learned social competencies and were more focused on people than before [22,23,24]. This enabled them to be more easily and successfully adopted into a new family. In particular, we observed a decrease in excitability, apathy, insecurity and increase in sociability, attention and self-confidence with the progression of training sessions [22,23,24]. The implementation of the RandAgiamo project led to some general improvement in all the dogs living in the shelter because all dogs could daily interact with staff and volunteers attending the shelter [22,23,24]. The RandAgiamo project acted as a social and environment enrichment program for all shelter dogs, promoting good social relationships among dogs, thereby fatal aggressions between cohabitant dogs were reduced to zero [23,24].

Several studies have shown the positive effect of human contact on the welfare and behaviour of dogs living in shelters [18,19,20]. Conley *et al.* [28] found that an increase in human contact reduced fear reactions in dogs, but this had no effect on their adoptability. On the contrary, Wells *et al.* [29] demonstrated that social contact with humans could increase the chance of adoption of shelter dogs. The amount of time spent with humans (familiar and unfamiliar) had a positive influence on the behaviour of shelter dogs [28,30]. Hennessy *et al.* [31] evaluated the impact of prison socialization programs on the adoption success of shelter dogs. Dogs participating in these programs were more responsive to trained commands and calmer in novel situations. Since behavioural problems, such as hyperactivity, excessive barking, destructiveness, uncontrollability and escape behaviour were a recognised cause of relinquishment of dogs to shelters [3], as well as lack of training [3,12], these positive modifications in dog behaviour might also affect adoption success. Socialization projects involving students, such as the “*Applied Animal Behaviour course*” of the Colorado State University, has also had great benefits for both students and dogs [32]. In Northern Italy, some shelters have introduced a social enrichment program, called “*Temporary Adoption Program*”, consisting of people regularly visiting, walking, playing and petting shelter dogs. Dogs participating in this “non-conventional” form of adoption were more active and friendly with visitors, thus increasing their chances of adoption and decreasing the number of returns after adoption. Interestingly, positive behavioural effects were also noted in sheltered dogs not directly involved in the program [7]. These results are in close agreement with the current study. Official data provided by the Umbria regional health authorities was insufficient to estimate the number of treated dogs (RandAgiamo dogs) that have been adopted. However, the activation of the RandAgiamo project involved a constant presence of RandAgenti and volunteers inside the shelter, acting as social enrichment even for the dogs who remained untrained. In fact, in practice, dogs were able to take advantage of all RandAgiamo activities even without being directly involved as part of the project. Indeed, the number of adopted dogs in the RandAgiamo shelter (206 dogs) exceeded the number of dogs actually treated (58 RandAgiamo dogs), suggesting that all the animals housed in the RandAgiamo shelter have benefited from the project. In a shelter in Vienna (Austria), a similar program of socialization was implemented, where dog lovers, called “*sponsor of care*”, took shelter dogs for walks [33]. In a few years, the length of stay significantly decreased [33].

Gacsi *et al.* [34] assumed that also short periods of human contact evoke attachment behaviour in rescued dogs, suggesting that these dogs living in poor social conditions show a kind of sensitization to social contacts. Then, non-regular contacts with the volunteers could promote detrimental effects on shelter dogs. However, the interruption of the “*Temporary Adoption Program*” had no significant detrimental effects on socialised shelter dogs [7]. Hewison *et al.* [35] investigated the effects of free and restricted visitor access in a shelter, where contact with visitors was unpredictable and limited because dogs were confined and could not physically interact with them. Findings highlighted a short-term improvement in welfare when visitors were prohibited, although physiological parameters did not differ. These findings suggest contact inside the shelter between humans and dogs should be well planned. An over-stimulating environment or prolonged interruptions to the human–dog relationship could be stressful and the inability to interact may induce frustration in dogs [35]. Howard and DiGennaro Reed [36] emphasized the importance of training and integrity of the volunteers implementing enrichment programs in shelters. The RandAgiamo protocol respected these precepts providing a systematic planning of sessions, as well as a high level of commitment by well-trained volunteers. In addition, great efforts were made to avoid dog attachment problems (and sometimes also staff attachment problems) by rotating the RandAgenti and volunteers in the training and walking of each dog. However, long-term evaluation of the bond between shelter dogs and trainers should be investigated.

The nature of the official data analysed in this study did not allow us to detangle the effects of the individual training from other elements, such as social contact and publicity. However, these were key elements of all the RandAgiamo philosophy, which was strongly focused on changing public opinion on shelter dogs’ characteristics. It was important for the project to demonstrate that shelter dogs were well trained, well behaved in daily life situations to demolish the prejudice that they are not good dogs, or have undesirable behavioural problems. Therefore all RandAgiamo dogs were behaviourally assessed as part of the programme and subsequently trained by the RandAgenti to ensure each dog acquired socially acceptable behaviours. RandAgiamo training has previously proved to be effective in changing dog behaviour [22,23,24]. It was found that in shelter management practice this was one of the most potent tools to increasing dog adoptability, because the potential adopters were confident these dogs were reliable and not problematic. In agreement with Luescher *et al.* [20], current findings might indicate that training could also have a positive effect on shelter dogs’ behaviour and welfare. Several authors found that dogs attending training courses were more attractive to potential adopters and less likely to return to shelters [3,14,20,37]. According to King *et al.* [38], in the opinion of a potential adopter, the way that a dog behaves is more important than its physical appearance, and the most desirable traits included being a well housetrained, sociable, and obedient dog. Other authors suggest that training courses for shelter dogs could improve the characteristics considered as most important for owners [20,39]. Human contact and training programmes have been shown to increase the time dogs spend standing at the front of their pens, making them more attractive to visitors, thus increasing their chance of being adopted [28,29]. Previous studies found that attending obedience training was associated with a reduced prevalence of behavioural problems and increased canine sociability [14,39,40]. Some authors found that training increased desirable behaviours in shelter dogs, such as sitting, being quiet, making eye contact, while it decreased undesirable behaviours, such as jumping [21,41]. However, a direct impact on adoption rate or length of stay was not observed [21,41]. Conversely, Luesher *et al.* [20] found that shelter trained dogs were 1.4 times more likely adopted than untrained ones. The current findings show that in the shelter where the RandAgiamo project was used dogs were three times more likely to be adopted.

In other studies, the introduction of environmental enrichments in dog shelters, ranging from music or new odours to cage furniture, showed a positive effect on dogs’ welfare [28,42,43,44]. Besides increasing behaviours considered most desirable by potential adopters, such as being calm and sociable, these enrichments made shelters more attractive, influencing visitors to adopt a dog. This also suggests that the shelter environmental context should be valorised to increase adoption rates [29]. In the RandAgiamo and the Control shelters, animal husbandry was managed by the same animal protection organization. Inanimate enrichment or cage furniture did not vary significantly between the two shelters. However, in the RandAgiamo shelter, some variations to the daily animal management was adopted in accordance with the philosophy of the project, such as giving the dogs the opportunity to range free in an outdoor common space most of the day, promoting inter-specific social interactions.

Moreover, the increased adoption rate at the RandAgiamo shelter may also be due to the initiatives increasing the visibility of dogs out of the shelter, such as promotion in shopping malls and public gardens, newspapers and social media. These complementary activities acted as a campaign to raise public awareness on the problem of stray dogs and gave some RandAgiamo dogs the chance to meet the general public in daily life. The “*foster programs*”, as described by Mohan-Gibbons *et al.* [6], considered the reluctance people have in acquiring dogs from shelters, so they placed adoptable shelter dogs in foster homes, called “*Adoption Ambassadors*”. These dogs were walked in public areas to make them visible and familiar to potential adopters. These foster home programs obtained encouraging results bringing benefits both to the welfare of dogs and to the shelters’ budget [6]. In the current paper, we analysed the synergic effect of all the elements implemented by the RandAgiamo project (including social contact, training, and publicity) to increase dog visibility and adoptability. The RandAgiamo project has been evaluated as a whole, considering the implementation of all planned activities. Moreover, it was not possible to separate the effect of individual elements of the project due to the lack of specific data. Therefore, further study will be carried out to better evaluate the individual influence of each element of the project.

Some authors report that to increase adoption rates it is important to implement effective methods for selection of shelter dogs suitable for adoption [45], and to provide basic education on dog behaviour and physiology for potential adopters [6,46]. Pre-adoption counselling has included a description of common signs of separation anxiety and advice on how to manage behavioural problems, information on dog reproductive physiology, and breed characteristics [6,46]. An increase in the knowledge of potential adopters may have contributed to the success of the RandAgiamo project in a similar manner to the “foster programs” [6]. However, when pre-adoption counselling is insufficient and generic, it is ineffective [47].

Another element of the RandAgiamo project was the post-adoption assistance. This program enabled adoption with a “warranty” of sorts, not only for the new owner, but also for the dog, whose well-being was always a priority. Owners felt assured by taking a RandAgiamo dog because they could rely on the free consultancy by RandAgiamo staff if any behavioural problems occurred after adoption. In a post-adoption survey, the RandAgiamo owners reported a high level of satisfaction with their dogs, who they considered desirable, well-educated and socially integrated with their family [22,23]. This is of utmost importance because training shelter dogs not only promoted their chances of being adopted but also made them less likely to be re-abandoned or returned to the shelter, because they were happily bound to their new owners. This was very important considering that the RandAgiamo project is designated mainly to adult dogs, that usually have less chance of being adopted and a higher chance of returning to a shelter [1,3,7].

In the RandAgiamo shelter, we recorded the highest restitution rate of captured dogs to their owners. This may be due to the location of this shelter in the capital of the province (Perugia). Perhaps, people living in the city are more aware of the importance of enrolling their dogs in the National Dog Register Service compared with people living in small villages or in the country. Registration allowed owners to identify their lost dogs, and they could be returned to them. This may reflect differences in the attitude towards animals and the human–animal bond in relation to the living environment [3,38].

Overall, the current findings suggest that rescue shelters had adoption rates greater than rehoming ones. It is not easy to provide an explanation for this. It may be due to different management frameworks, *i.e.*, rescue shelters are under the supervision of public veterinary services, whereas rehoming shelters are run by private animal protection organizations. In the rehoming shelters, dogs tended to remain for a longer time possibly leading to behavioural problems which may compromise their adoptability [14]. Moreover, in rescue shelters, dogs might be more visible due to owners searching for their lost dogs. However, this result is unexpected because all puppies are usually housed in rehoming shelters and are the most desired dog by potential adopters [38].

There is much more at stake than just public cost in the current system of dog shelters in Italy. In the essence of the RandAgiamo philosophy, it is an ethical and animal welfare issue, in addition to having a social and financial impact. As already mentioned, most of the RandAgiamo staff worked on a voluntary basis, which made this program economically affordable. However, further studies analysing the cost-benefits of future projects should be carried out to help regional authorities make decisions on the suitability of the RandAgiamo model on a large scale.

## 5. Conclusions

The RandAgiamo pilot project implemented in a rescue shelter of the Umbria Region was successful. Several elements may have contributed to the increase of adoptions in the shelter: (i) improvement of dogs’ behaviour making shelter dogs more attractive to potential adopters; (ii) advertising and promotion of shelter dogs in public areas and social media, giving greater visibility and changing public perception; and (iii) pre- and post-adoption assistance that reassured new owners and ensured their satisfaction. However, the individual influence of each element of the RandAgiamo protocol (social contact, training, and publicity) needs to be further assessed.

This program provided social enrichment leading to many benefits for both the shelter and the dogs, including those dogs not directly involved. However, well-trained staff were necessary to provide training and socialization to shelter dogs and promote adoption related activities. Further studies are needed to analyse the cost-benefits of the project, as well as to explain differences in adoption rates between rescue and rehoming shelters and the cultural and social reasons for dog restitution rates.

## Supplementary Files

Supplementary File 1
