# Supplementary material for: RandAgiamo™, a Pilot Project Increasing Adoptability of Shelter Dogs in the Umbria Region (Italy)
Source: Animals (Basel). 2015 Aug 14;5(3):774–92. doi: 10.3390/ani5030383 (PMC4598705; doi:10.3390/ani5030383)
Supplement: Supplementary File 1 [file animals-05-00383-s001.pdf]

Supplementary Material. Iter of a captured dog in Rescue and Rehoming shelters.

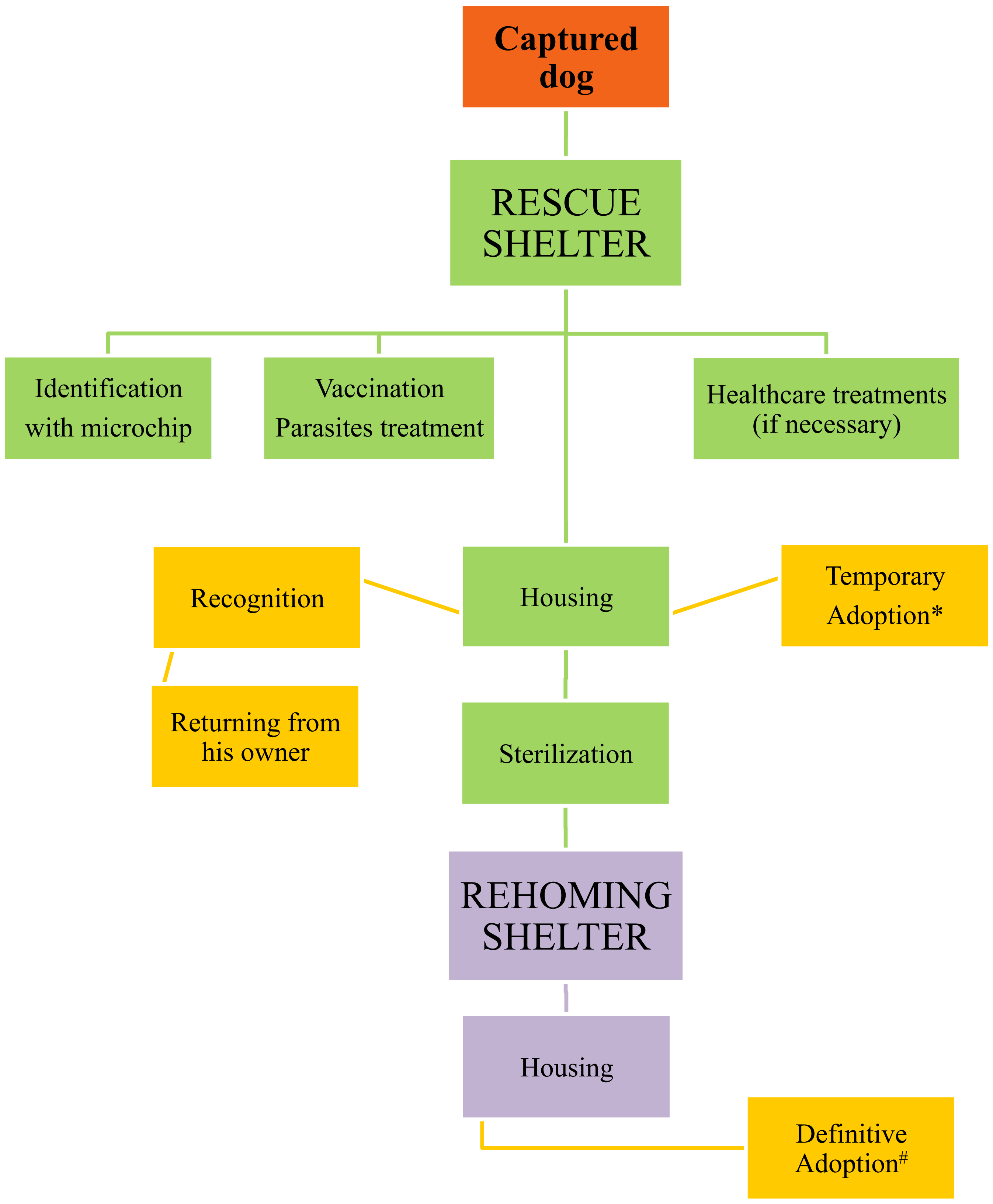

* Adoption from a rescue shelter is “temporary” if made within 60 days after dog capture. If the dog is not recognized and reclaimed by its owner within this time, the adoption can became “definitive”.

Adoption from a rehoming shelter is always “definitive”
